# Differentiation of human adipose-derived stem cells into neuron/motoneuron-like cells for cell replacement therapy of spinal cord injury

**DOI:** 10.1038/s41419-019-1772-1

**Published:** 2019-08-08

**Authors:** Shane Gao, Xuanxuan Guo, Simeng Zhao, Yinpeng Jin, Fei Zhou, Ping Yuan, Limei Cao, Jian Wang, Yue Qiu, Chenxi Sun, Zhanrong Kang, Fengjuan Gao, Wei Xu, Xiao Hu, Danjing Yang, Ying Qin, Ke Ning, Pamela J. Shaw, Guisheng Zhong, Liming Cheng, Hongwen Zhu, Zhengliang Gao, Xu Chen, Jun Xu

**Affiliations:** 1 East Hospital, School of Medicine, Tongji University, Shanghai, 200120 China; 2 iHuman Institute, Shanghai Science and Technology University, Shanghai, 201210 China; 3 Shanghai Public Health Clinical Center, Fudan University, JinShan Shanghai, 201508 China; 4 Department of Neurology, Third Affiliated Hospital of Navy Military Medical University, Shanghai, 200438 China; 5 Tongji hospital affiliated to Tongji University, Tongji University School of Medicine, Shanghai, 200065 China; 6 Shanghai Eighth People’s Hospital Affiliated to Jiangsu University, Shanghai, 200233 China; 7 Department of Orthopaedics, Shanghai Pudong Hospital, Fudan University Pudong Medical Center, Shanghai, 200137 China; 8 Zhoupu hospital, Affiliated to Shanghai University of Medicine & Health Sciences, Shanghai, 201318 China; 9 Department of Neuroscience, Sheffield Institute for Translational Neuroscience (SITraN), University of Sheffield, 385A Glossop Road, Sheffield, S10 2HQ UK; 10 Tianjin Hospital, Tianjin, 300211 China; 11 BOE Technology Group Co., Ltd., Beijing, 100176 China; 12 Tenth People’s Hospital, School of Medicine, Tongji University, Shanghai, 200092 China

**Keywords:** Neurogenesis, Stem-cell research

## Abstract

Human adipose-derived stem cells (hADSCs) are increasingly presumed to be a prospective stem cell source for cell replacement therapy in various degenerative and/or traumatic diseases. The potential of trans-differentiating hADSCs into motor neuron cells indisputably provides an alternative way for spinal cord injury (SCI) treatment. In the present study, a stepwise and efficient hADSC trans-differentiation protocol with retinoic acid (RA), sonic hedgehog (SHH), and neurotrophic factors were developed. With this protocol hADSCs could be converted into electrophysiologically active motoneuron-like cells (hADSC-MNs), which expressed both a cohort of pan neuronal markers and motor neuron specific markers. Moreover, after being primed for neuronal differentiation with RA/SHH, hADSCs were transplanted into SCI mouse model and they survived, migrated, and integrated into injured site and led to partial functional recovery of SCI mice. When ablating the transplanted hADSC-MNs harboring HSV-TK-mCherry overexpression system with antivirial Ganciclovir (GCV), functional relapse was detected by motor-evoked potential (MEP) and BMS assays, implying that transplanted hADSC-MNs participated in rebuilding the neural circuits, which was further confirmed by retrograde neuronal tracing system (WGA). GFP-labeled hADSC-MNs were subjected to whole-cell patch-clamp recording in acute spinal cord slice preparation and both action potentials and synaptic activities were recorded, which further confirmed that those pre-conditioned hADSCs indeed became functionally active neurons in vivo. As well, transplanted hADSC-MNs largely prevented the formation of injury-induced cavities and exerted obvious immune-suppression effect as revealed by preventing astrocyte reactivation and favoring the secretion of a spectrum of anti-inflammatory cytokines and chemokines. Our work suggests that hADSCs can be readily transformed into MNs in vitro, and stay viable in spinal cord of the SCI mouse and exert multi-therapeutic effects by rebuilding the broken circuitry and optimizing the microenvironment through immunosuppression.

## Introduction

Spinal cord injury (SCI) is a devastating condition and often results in life-long consequences such as paralysis and associated complications^1^. A cascade of secondary events follow the initial traumatic injury, characterized by ischemia, hemorrhage, edema, inflammation, cell death, and further tissue damage, and eventually result in neuron demyelination, axonal degeneration, and cavitation at the injury site^2–5^. Existing treatments, include surgery and antibodies against myelin-associated neurite growth inhibitor^6^ and neurotrophic factor protection^7,8^, aiming to stabilize the injury and prevent secondary complications. However, these strategies have limited therapeutic benefits presumably due to their reliance on the adult body’s limited self-repairing capacity to recover and rebuild the disrupted neuronal network.

Stem cell therapy, as an alternative strategy, holds great promise for spinal cord injury treatment for the ability of stem cells to self-renew, to differentiate into multiple lineages^9,10^, to secrete neurotrophic factors^8,11,12^ and anti-inflammatory cytokines^13–17^. Several stem cell-based strategies have been tested in experimental and clinical settings for therapeutic effects in SCI therapy but with variable hurdles^14–16,18–21^.

Mesenchymal stem cells (MSCs) are a group of heterogeneous multipotent adult stem cells^22,23^ can be differentiated into chondrocytes^24,25^, adipocytes^26^, myocytes^27,28^, endothelial cells^29,30^, and neurons^31–34^. The main advantages of adipose-derived stem cells (ADSCs), include the feasibility of autologous transplantation, the ability to secrete neurotrophic factors and cytokines to promote the survival of endogenous cells, and suppress the inflammatory response to facilitate tissue repair^17,35,36^.

Among various MSCs, ADSCs take many advantages over bone marrow stem cells, such as easier to harvest, low senescence, and rich in tissue^37^. ADSCs may be pre-programmed to differentiate toward a neural lineage^38^, which has been confirmed by several reports^39–44^. ADSCs are also capable of supporting oligodendrocytes in vivo^45^ and transforming into functional Schwann cells^46–50^.

Since loss of motoneurons is a main cause of permanent disability in SCI patients, it is expected that replacement of motoneurons and/or prevention of secondary complications to the remaining motoneurons may lead to some functional recovery. Mouse and human ESC-derived motoneurons transplanted into animal models of SCI could survive and integrate into the lesion site with significant recovery of motor function^51,52^. Recently, two research groups independently reported the induction of hADSCs into motoneuron-like cells^40,53^, using a protocol modified from that used to convert ESCs/iPSCs into motoneurons^54,55^. However, the therapeutic potential of hADSC-derived motor neuronal cells has yet to be established in a clinic relevant SCI model. This study for the first time optimized a stepwise protocol for high efficient ADSC-motoneuron trans-differentiation and further investigated the therapeutic potential of hADSCs-MN for SCI. We proved that pretreated hADSCs could survive, integrate and become electrophyisiologically active neuronal cells in injured spinal cord and participate in re-establishing the broken neural circuits through a relay fashion as well as optimizing the microenvironment by immune-suppression, which eventually led to functional recovery in locomotor.

## Results

### Differentiation of hADSCs into electrophysiologically active motoneuron-like cells in vitro

**Fig. 1:**
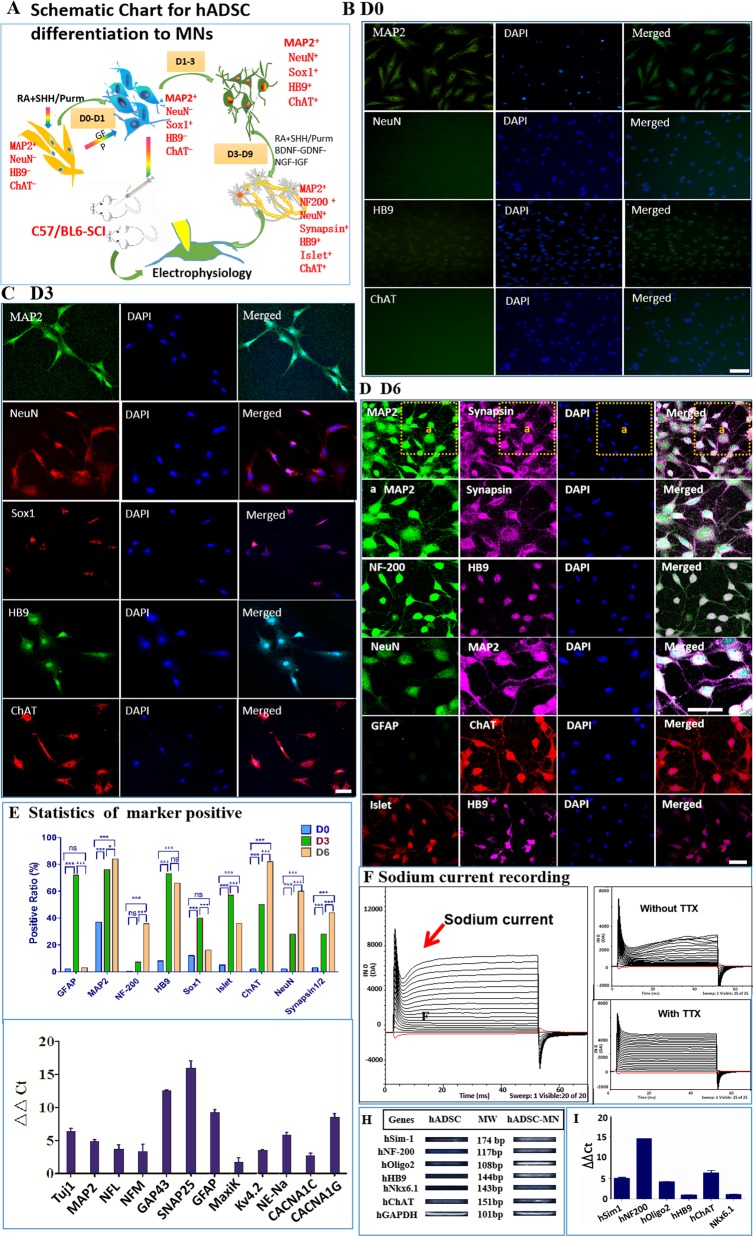
Differentiation of hADSCs into motoneuron-like cells by a step-wise protocol **a** Schematic flow chart for in vitro differentiation of hADSCs into motoneuron-like cells. **b** Before induction, hADSCs have little expression of neuronal or motoneuron markers such as NeuN, HB9, ChAT, except to low expression of MAP2. **c** After 3 day induction with Purmorphamine(or SHH) and RA, hADSCs start to express high level of neuron/motoneuron markers MAP2, NeuN, HB9, ChAT, as well as motoneuron progenitor marker Sox1. **d** Another 3 days’ induction supplemented with neurotrophic factors further boosts the maturation of the hADSC-derived motor neuron-like cells with the high-level expression of neuron/motoneuron markers MAP2, NeuN, NF-200, Synapsin1/2, Islet, HB9, and ChAT. The neurites of the differentiated cells become more complex. **e** Quantification of immunostaining for various markers at different stages. **f** Sodium currents can be recorded at Day 6 with whole-cell patch-clamp, which can be blocked by 1 µM TTX. **g** The expression of various neuronal marker genes, including sodium, potassium, and calcium channels is elevated after 6 days’ induction by qRT-PCR. **h**, **i** The augmented expression of some motoneuron specific genes expression is verified by qRT-PCR. Scale bar:100 µm. Statistics is done by two-way ANOVA of Graphpad Prism 5

The experimental diagram is shown in Fig. 1a. The characterization of hADSCs was shown in Supplementary Fig. 1. SHH and RA are important for neuralization and ventralization of ESCs and iPSCs during neuron development^56–58^. Thus, we attempted to treat hADSCs with SHH and RA to initiate their differentiation toward motoneurons (Fig. 1b–d). Before induction, the hADSCs had only weak expression of neuronal marker MAP2 as well as the motoneuron marker HB9 without expression of any other neuronal progenitor markers of Olig2, Sox1, mature motor neuron markers of NeuN, ChAT, and Islet (Fig. 1b and Supplementary Fig. 2A). As early as 6 h after induction, morphological changes became noticeable. Afterward, hADSCs gradually became polarized and formed neurite-like structures. By day 3, hADSCs had already expressed various neuronal progenitor markers, such as GFAP, Sox1, Olig2, as well as motoneuron markers of NeuN, Synapsin1/2, Islet, HB9, and ChAT (Fig. 1c and Supplementary Fig. 2B). Most of these differentiated cells were structurally simple and usually bi-polarized, and the expression of MAP2, ChAT, and Synapsin1/2 was mainly concentrated within soma implying that they were likely just turning into immature neurons/motoneurons without synaptic structures (Supplementary Fig. 2B).

Neurotrophic factors were supplemented to further accelerate the maturation of these nascent motoneuron-like cells for another 3–6 days. By day 6, complex dendritic structures with secondary or tertiary branches as well as puncta-like synapses were easily detectible. The expression of mature neuronal markers, such as MAP2, NF-200, NeuN, Synapsin1/2, and NF-200; as well as motoneuron markers HB9, ChAT, and Islet were significantly augmented (Fig. 1d and Supplementary Fig. 2C). On the contrary, the expression of progenitor markers such as Olig2, Sox1, and GFAP decreased dramatically (Fig. 1d, e, Supplementary Fig. 2C), indicating that the majority of the hADSCs were converted into relatively mature neurons/motoneurons with high purity by the protocols we developed (Fig. 1e).

To check whether hADSC-MNs could obtain action potential, whole-cell patch-clamp recordings were done after 6-day induction. In the whole-cell voltage-clamp configuration, voltage-dependent inward currents were recorded from hADSC-MNs (Fig. 1f). When 1 µM TTX was added, these inward currents were completely blocked, indicating that these inward currents were typical voltage-gated sodium currents (Fig. 1f). To verify these findings, qRT-PCR data demonstrated that voltage-dependent sodium, potassium, and calcium channels were expressed in these motoneuronal-like cells. (Fig. 1g). Some lineage-specific markers such GAP43, an interneuronal marker, and GFAP, a neural stem cell or glial marker, were detectable (Fig. 1g). The expression level of motoneuronal and synaptic marker genes, such as SIM1, OLIG2, HB9, CHAT, NKX6.1, NF-200, and SNAP25, were all upregulated (Fig. 1h, i). Taken together, our step-wise protocol could transdifferentiate hADSCs to electrophysiologically functional neurons.

### Therapeutic effects of the transplanted hADSC-MNs in the SCI mouse model

**Fig. 2:**
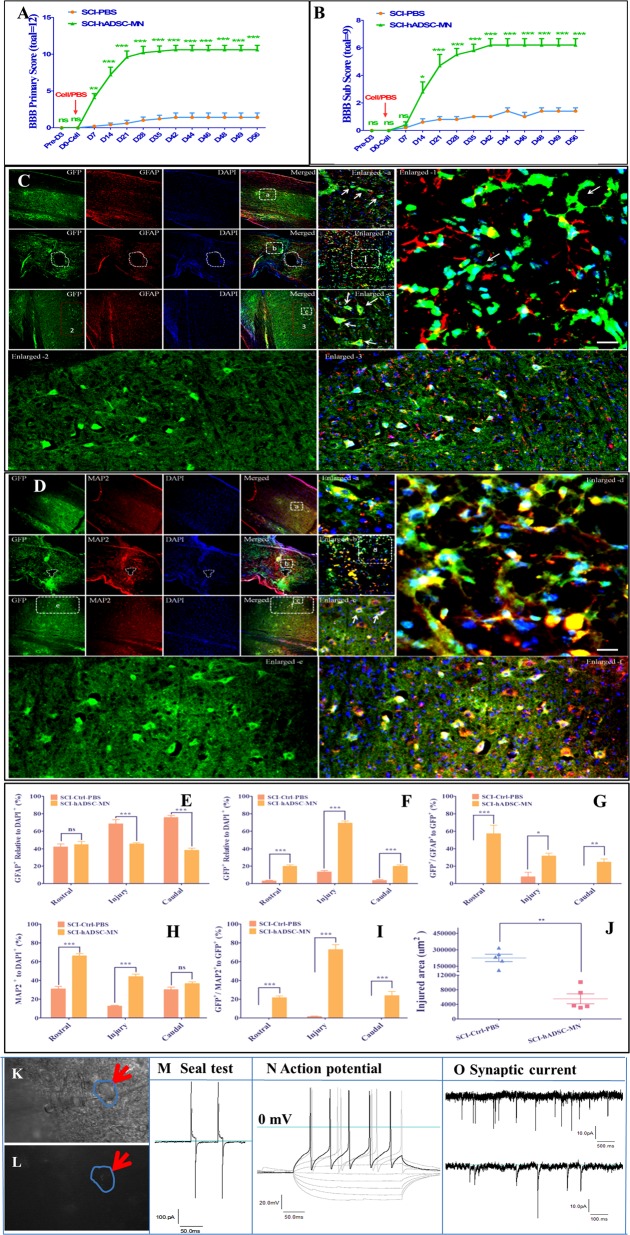
Transplanted hADSCs survive, integrate, and differentiate into functional neurons at the injured spinal cord in SCI mouse models. **a**, **b** Transplantation of preconditioned hADSCs promotes the recovery of locomotor capacity of SCI mice as evaluated by the BMS scoring (both primary and subscore, *n* = 12) at different time points. **c** Immunostaining of spinal cord slices from SCI mice with the astrocyte marker GFAP 8 weeks after hADSC transplantation. Representative images from the rostral, central, and caudal part of the injury site are shown in enlargements **a–c** as well as further enlargement 1–3, respectively. **d** Immunostaining of spinal cord slices from SCI mice with the neuronal marker MAP2 8 weeks after hADSC transplantation. Representative images from the rostral, central, and caudal part of the injury site are shown in enlargements **a**–**c**, respectively; scale bar: 400 and 50 μm (for the enlarged pictures). **e**–**g** Statistics for the ratio of GFAP positive astrocytes out of the total cell count, GFP-positive transplanted cells out of the total cell count and the GFAP-positive astrocytes derived from GFP-positive transplanted cells respectively, at various sites (*n* = 5). **h**, **i** Statistics for the ratio of MAP2-positive neurons out of the total cell count, MAP2-positive neurons out of the total transplanted cells (*n* = 5); **j** the area of cavitation at the injury site was calculated with Image-Pro Plus software (*n* = 12). Quantification was performed by counting at least five fields per sample for each slice and analyzed by student *t* test; **k**–**o** patch-clamp whole-cell recording is performed on the GFP-labeled hADSC-derived neuron-like cells in the acutely prepared spinal cord slice from SCI mice. **k**, **l** Representative bright field image of a patched cell with fluorescence illumination; **m** a representative trace shows that the good seal (GΩ) can be achieved; **n**, **o** the representative traces of action potentials and spontaneous synaptic currents, respectively, recorded from transplanted GFP-positive hADSCs

Whether hADSC-MNs could exert therapeutic effects in vivo in a rodent SCI model was unknown. The schematic experimental plan was as shown in Supplementary Fig. 3A. A T8 crushed SCI mouse model was developed^59^ was shown in Supplementary Fig. 3B, C. Transplanted hADSC-MNs successfully decreased injury cavity as shown in Supplementary Fig. 3D. Basso, Beattie, Bresnahan-derived Basso Mouse Scale (BMS) locomotor rating scale scores in total (Supplementary Fig. 3E), including the primary score and subscore, were applied and processed (Fig. 2a, b). The scores of the phosphate-buffered saline (PBS) control group increased gradually and reached a plateau around 3 weeks and last until the end of the observation after the injury, while hADSC-MN group increased steadily and continuously up to 5 weeks before reaching a plateau which was significantly higher than those achieved by the PBS control group (*P* < 0.001). The transplanted hADSC-MNs appeared to exert a positive-therapeutic effect as early as 1-week post-transplantation (*P* < 0.05) (Fig. 2a, b). These data demonstrate that hADSC-MN transplantation can significantly ameliorate the histopathological, behavioral, and functional deficits generated in this SCI mouse model.

### The survival and integration of transplanted hADSC-MN into the injured spinal cord

Next, we performed immunohistochemical staining to determine the fate of the transplanted cells. As expected, no GFP-positive cells were detected in the PBS control group. The injury site remained visible with obvious cavity (Supplementary Fig. 3D, F, and G). In contrast, a large number of GFP-positive cells were observed in the hADSC-MN transplanted group, mostly in the center of the injury site and the rostral and caudal surrounding areas bilaterally (Fig. 2c, d). The GFP-positive cells were predominantly (>80%) MAP2-positive but occasionally GFAP positive (<10%), suggesting that the transplanted hADSC-MN mainly differentiated toward a neuronal lineage in vivo (Fig. 2e–i). Furthermore, the preconditioned hADSCs adopted a multipolarized morphology in vivo resembling mature neurons, appeared to integrate with the host tissue and migrated out for at least several millimeters from the site of injection (Fig. 2c, d, enlarged 1–3 and a–c). The enlarged showed the caudal part away from the injury center. The sizes of the cavities that formed after injury were significantly smaller in the transplanted group compared to the control group (Fig. 2j). Most importantly, it is intriguing to explore whether the transplanted cells can integrate into the injured site of spinal cord and become electrophysiologically functional. Indeed, GFP-labeled hADSC-MNs were subjected to the whole-cell patch-clamp recording from acutely prepared slices of the injured spinal cord and demonstrated the capacity of firing action potential and receiving spontaneous synaptic inputs (Fig. 2k–o), which further demonstrates the long-term viability, success of neural conversion and functional integration of the transplanted human cells into the host spinal cord tissue.

### The transplanted hADSC-MNs directly participate in re-establishing the broken neural circuitry in injured site

**Fig. 3:**
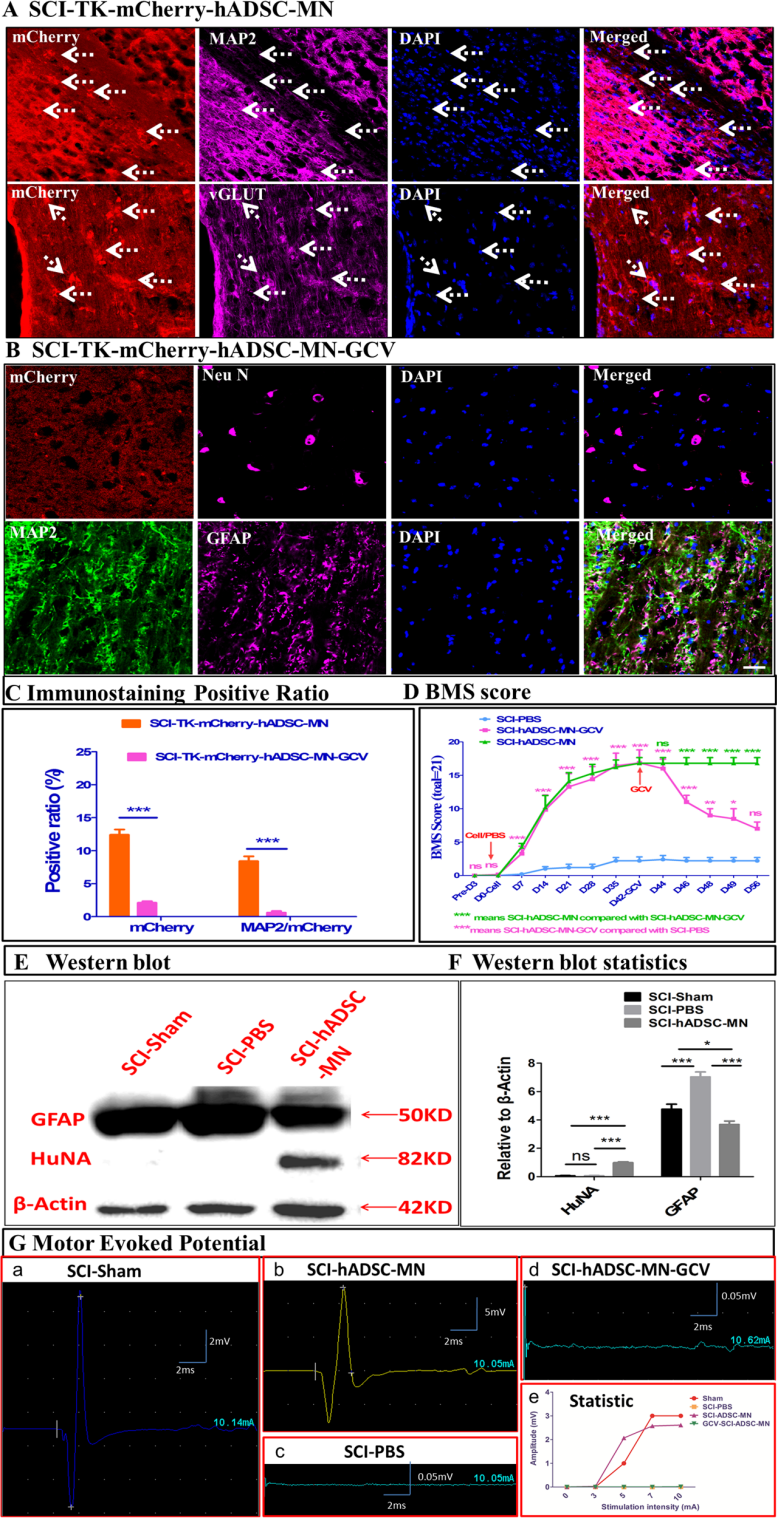
The transplanted hADSCs can participate in reconstitution of the broken circuitry and lead to the behavioral recovery of the SCI mice. **a** Immunostaining images demonstrate hADSCs are effectively infected by HSV-TK-mCherry-GCV viruses and become neuronal-like cells and integrate into the injured site of SCI spinal cord, as stained positively by MAP2 and vGLUT. **b** Administration of GCV can eliminate most of the transplanted cells as demonstrated by co-staining of mCherry with NeuN, MAP2, and GFAP. **c** Statistic analysis shows both mCherry-positive cells and mCherry-positive neurons are largely deleted by GCV administration within 2 weeks (*n* = 5); **d** BMS scoring in total demonstrate that GCV administration leads to gradual worsening of locomotor capacity and functional relapse (*n* = 12); **e** Western blotting data show positive expression of HuNA in the SCI-hADSC-MN group, further demonstrating the long-term survival of transplanted cells; **f** Western blotting quantification data is analyzed by two-way ANOVA; **g** motor-evoked potential is analyzed among different groups to test the integrity of the neural circuitry along the corticospinal axis, showing that the transplanted hADSCs are essential for the re-establishment of neural circuitry in SCI mice and deletion of transplanted cells by GCV will lead to the disruption of the neural circuitry again (*n* = 12). Scale bar: 25 μm

**Fig. 4:**
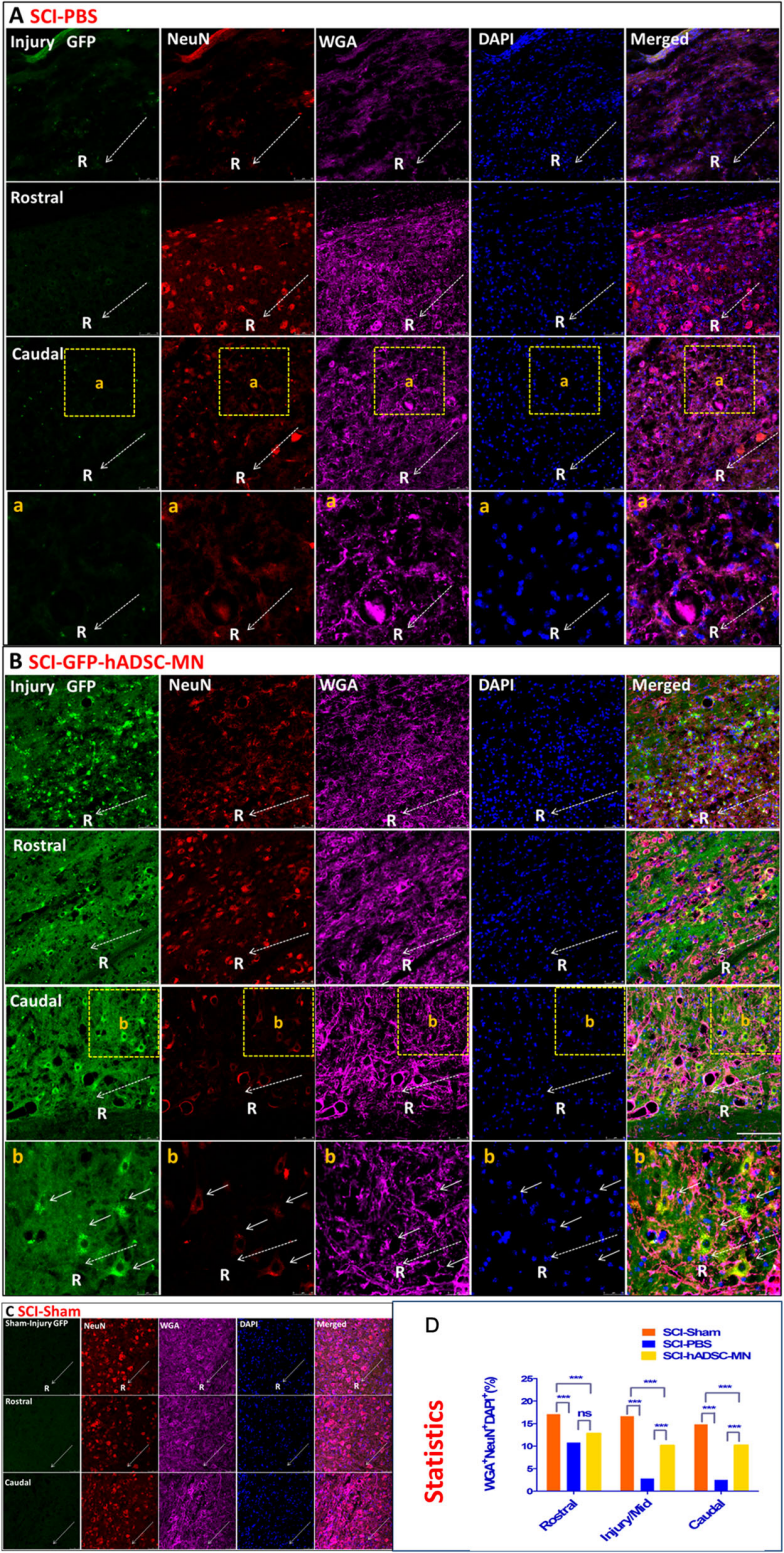
Functional rebuilding of neuronal circuitry by transplanted hADSCs in SCI mice as revealed by retrograde tracing marker WGA. The data show the immunostaining with WGA and NeuN antibodies in the spinal cord slices from various groups. **a** The negative PBS control group; **b** the hADSCs transplanted group; **c** the Sham group; in each group, double staining of WGA and NeuN are performed and representative images from central, rostral, and caudal part around the injured site are shown, demonstrating the morphological and functional integration of hADSC-MN into the host spinal cord tissue; **d** statistics of the WGA-, NeuN-, and DAPI-positive ratio from each site for various groups of mice (*n* = 5), Scale bar: 100 μm

To check whether the introduced hADSC-MNs can functionally integrate into the neuronal circuitry, HSV-TK-mCherry-Ganciclovir (GCV) cell suicide system was applied^60^. The BMS scoring data indicated that after 8 days of continuous GCV injection per day, the BMS score gradually but significantly decreased, implying the functional relapse of the mobility capacity of injured mice (Fig. 3d). Before the administration of GCV, the mCherry-labeled hADSC-MNs were easily detectible at the injured site and could be co-stained by neuronal marker MAP2 (Fig. 3a). After GCV injection, the mcherry-positive cells sharply decreased compared with the non-GCV injected counterpart (Fig. 3b, c). Western blotting data demonstrated the human specific nuclear antigen was expressed in the hADSC-MN transplanted group (SCI-hADSC-MN) and expressed neither in the group of SCI-Sham nor the group of SCI-PBS, implicating the long-lasting existence of transplanted human cells in vivo (Fig. 3e, f). We further performed the in vivo electrophysiological experiment to test the integrity of the cerebrospinal neural circuits under various conditions. The motor-evoked potentials (MEP) were elicited in the frontal cerebral cortex and recorded in the skeletal muscle of hind limb of the mice. MEPs could be steadily recorded before the surgical interference (Fig. 3g, inset a). After establishing the SCI model, the MEP could be barely recorded which could be restored by hADSC-MN transplantation (Fig. 3g, inset b and c). After GCV administration, the MEPs were largely abolished again (Fig. 3g, inset d and e), indicating the essential role of transplanted cell in ensuring the conduction of electric signals. Altogether, these data demonstrated that the transplanted hADSC-MNs survived, integrated, and directly participated in rebuilding the disrupted neural circuitry in SCI model. To determine how the transplanted hADSC-MNs established the synaptic connection with endogenous neurons, retrograde neuron tracing with wheat-germ agglutinin (WGA) was carried out^61^. Significantly, higher expression of WGA was observed at the center of the injured site as well as the caudal part adjacent to the injured site, implying the re-establishment of the synaptic connections across the injured site of the spinal cord (Fig. 4a–d). WGA had been detected in large amount of transplanted hADSC-MNs as indicated by co-localization of GFP with WGA (Fig. 4b, inset b), suggesting that endogenous neurons directly innervate the transplanted cells with synapses.

### Immunosuppressive effect of transplanted hADSC-MNs at the injured site of spinal cord in SCI mice

**Fig. 5:**
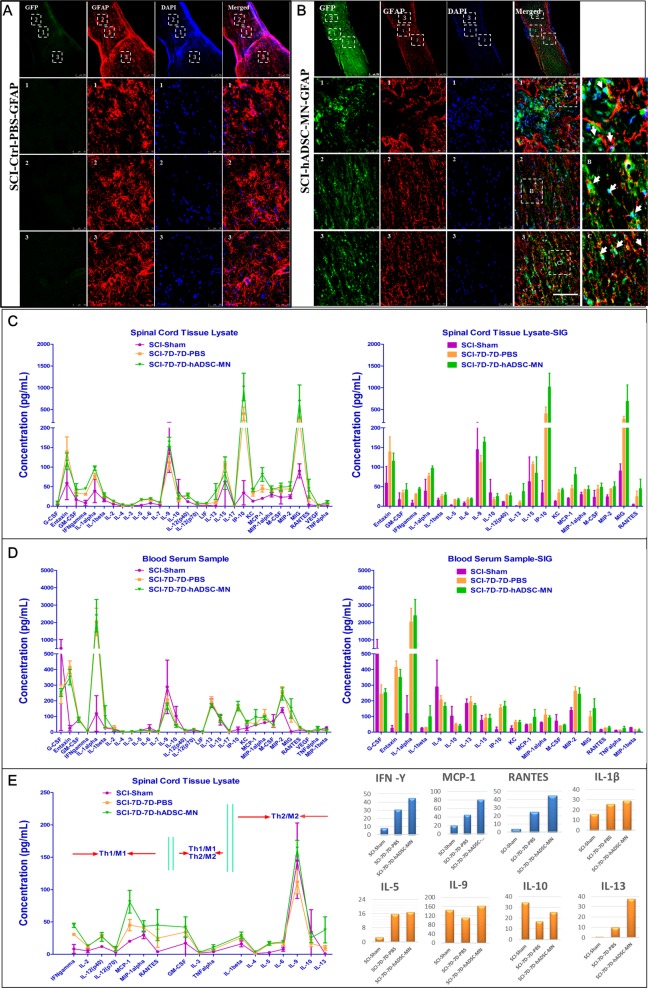
Transplanted hADSC-MNs exert suppressive effect on the inflammatory response in SCI mouse models. **a** In the injury site, striking glial activation was present in the PBS control group as revealed by high expression of GFAP. Scale bar: 250 and 50 μm (for A 1–3). **b** In the hADSC-MN transplanted group, GFAP expression was significantly attenuated by the presence of GFP-positive transplanted cells, demonstrating their anti-inflammatory effects. Scale bar: 250 and 50 μm (for B 1–3). **c**–**e** Immunocytokine secretion profiling in both spinal cord lysates and blood serum is analyzed in various groups; **c** the cytokine profiling in the spinal cord lysates; **d** the cytokine profiling in the blood serum; SCI mice injected with PBS or hADSC-MN 7 days after surgery and collected spinal cord lysate supernatant 7 days after injection was grouped as SCI-7D-7D-PBS and SCI-7D-7D-hADSC-MN, respectively, and the scramble group labeled as SCI-Sham. The experimental protocol strictly follows the MCYTOMAG-70K-PX32 (Merk Millipore, Lot#2618731); **e** the variation of cytokine profiling correlated with the corresponding macrophage and pro- or anti-inflammation factors changes; Scale bar: 100 μm

It is intriguing to test whether hADSC-MNs can exert immune modulatory effects at the injured site. As expected, SCI led to a massive inflammatory response with glial activation as indicated by numerous GFAP^+^ cells present and persisting in and around the injury site (Fig. 5a). This peri-lesional reactive gliosis was vastly reduced (Fig. 5b) in the hADSC-MN treated group, strongly suggesting that the transplanted hADSC-MN had a suppressive effect on the injury-induced inflammatory response. The immune factor profiling for both blood serum and the spinal cord lysate samples at 7 days after hADSC-MNs transplantation showed that the spinal cord injury caused a global inflammatory response as revealed by significantly increased level of pro-inflammatory factors, such as IL1α, MCP-1, RANTES, INF-γ, etc., both in the blood serum and cell lysates from the injured sites (Fig. 5c–e). However, the transplanted hADSC-MNs elicited a focal immunosuppressive effect at the injury site by upregulating the expression of a spectrum of anti-inflammatory factors, including IL-5, IL-9, IL-10, IL-13, etc. This effect is relatively restricted to the site where the transplantation was implemented since the immune factors in the serum did not change much by hADSC-MN transplantation.

## Discussion

Despite major advances in the medical and surgical care of SCI patients, so far no effective treatments have emerged to ameliorate the complications for severe SCI^5,62^. Stem cell-based therapy has the potential to tackle almost all the problems encountered in SCI, including loss of neurons and schwann cells, axonal degeneration and demyelination, disrupted vascular structures, and chronic inflammation^62–66^, and is an emerging approach with high potential for translation to the clinic. To date, various types of stem cells have been tested with variable recovery of function reported^20,67–69^. hADSCs are now increasingly considered the optimal choice of stem cells for various therapeutic applications due to their easy access, self-renewal ability, multipotency, free of immunogenicity^70,71^. In addition, in vitro neuronal differentiation of ADSCs has been reported by several research groups^12,49,72^ and the potential application of ADSCs for cell replacement therapy of various neurological diseases including SCI has been highlighted. ADSCs also have the immune modulatory capacity to reduce inflammatory response and improve the microenvironment^73–75^ to promote the survival of both the transplanted and the endogenous cells.

Transplantation of mouse/human ESC-derived motoneurons in a mouse model of SCI has been reported to lead to improved locomotor function^67,76–78^. To date, little is known about hADSCs’ capacity for motoneuron differentiation in vitro^40,53^. No data are available on the potential for hADSC-derived motoneuron for spinal cord injury in vivo. Ideally, hADSC-MNs may take theoretical advantages over undifferentiated hADSCs in cell replacement for treating SCI. In the present study, we developed a robust protocol for isolated high pure hADSCs consistent with previous reports^12,49,72^. These hADSCs expressed most of the embryonic stem cell markers, including SOX2, OCT4, c-MYC, and NANOG^70^, but at lower levels compared with ESCs, might confer hADSCs the self-renewal capacity and multipotency alongside with low-tumorigenic potential. It is of critical importance to evaluate the tumorigenic potential of hADSCs, especially after multiple passages. In the present study, no tumorigenesis was detected 6 months after hADSC-MN transplantation. The isolated hADSCs were then induced into motoneuron-like cells in a stepwise protocol using SHH and RA initially to promote neuralization and ventralisation of ESCs^70,79,80^. Upregulated expression of motoneuron specific markers Sox1, OLIG2, Islet, HB9, and ChAT, and general neuronal markers MAP2, NeuN, SYNAPSIN1/2, and NF-200 suggest that these cells consisted of a mixture of progenitor cells and motoneurons. Further conditioning with neurotrophic factors BDNF, GDNF, and IGF-1 for 3 days promoted the maturation of hADSC-MNs^56,79^. The expression of the MN progenitor marker of OLIG2 diminished and SYNAPSIN assumed a punctate distribution, suggesting the formation of synaptic structures and thus an increase in the maturation of terminally differentiated MNs. These MN-like cells eventually became electrophysiologically active, but remain immature with very high input resistance and depolarized resting membrane potentials. These cells may be still in an intermediate stage of maturation considering that differentiation of human ESCs into functionally mature motoneurons normally takes more than 6 weeks in vitro^81^. Actually before induction, there was weakly detectable expression of several neuronal differentiation marker genes, including MAP2, Sim-1, Olig2, NKX6.1, and NF-200 observed in our study. Taken together, these data have demonstrated the feasibility of differentiating hADSCs into cells with neuron/motoneuron characteristics.

It is noteworthy that hADSC-MNs survived in WT mouse spinal cord for at least several months, strongly indicating that hADSCs and their differentiated progenitors might be immune privileged cells as is the case for other MSCs^82^. Therefore, it is interesting to know whether ADSCs share similar immune properties, MHC I positive, MHC II negative, and lacking the co-stimulatory molecules CD40, CD80, and CD86^83^. Another important property of hADSCs is that these transplanted cells exert an anti-inflammatory effect as demonstrated by their ability to inhibit gliosis as well as the spinal cord immune cytokine profiling variance. The immune factor profiling pattern demonstrated that these hADSC-MNs could dramatically drive the tissue specific macrophage to transform from M1 type to M2 type, which may result from the accelerated IL-13 expression. Also, the transplantation of hADSC-MNs stimulates IL-9 expression in the spinal cord lysates. IL-9, through the interleukin-9 receptor (IL9R), activates different signal transducer and activator proteins and thus connects this cytokine to various biological processes such as stimulating cell proliferation and prevents apoptosis, inhibit TNF-α release^84^. The upregulation of IL-9 in spinal cord lysates of SCI-hADSC-MN group of mice compared with the SCI-PBS group of mice implied good survival of implanted hADSC-MNs and promotion of endogenous neuronal cells. MCP-1, also known as CCL2, recruits monocytes, memory T cells, and dendritic cells to the sites of inflammation produced by either tissue injury or pathogen infection^85^. Higher expression of MCP-1 in the spinal cord lysates of the SCI-hADSC-MN than SCI-PBS mice meant the transplanted hADSC-MNs could recruit inflammatory cells to the injured spinal cord and participate the SCI repair, and meanwhile, promote the anti-inflammatory factor expression of IL-5, IL-10, and IL-13. The immunosuppression of MSCs has been reported to result from the concerted action of chemokines, such as INF, TNF-α, and IL-1 with nitric oxide^74,75,86,87^. Pro-inflammation factors, including IFN-γ, MCP-1, RANTES, IL-1β, and anti-inflammatory factors, such as IL-10 and IL-13 were relatively higher in the spinal cord lysates of SCI-hADSC-MNs than the SCI-PBS groups. In addition, hADSCs have the ability to secrete various factors, including HGF, VEGF, TGF-β, IGF-1, interleukins, adiponectin, angiotensin, cathepsin D, pentraxin, pregnancy zone protein, retinol-binding protein, and CXCL12^88,89^, which can potentially inhibit apoptosis, promote vascular reconstitution, minimize inflammation and optimize the microenvironment at the injury site.

Evidence is needed for whether the therapeutic effect results from the replacement of degenerative cells by hADSC-MNs. Therefore, it is important to determine whether a functional relapse could happen if the transplanted cells are eliminated after stable recovery is established. Function relapse both from hindlimp locomotor behavior and muscle electrical potential were found on the HSV-TK-mCherry-hADSC-MN transplanted mice after i.p. injection of ganciclovir. In addition, analysis of the subtype fate of hADSC-MN is also crucial to clarify the therapeutic mechanism. It is likely that in the in vivo conditions, the terminally differentiated cells consist of motoneurons as well as other types of neurons, such as glutamatergic and GABAergic neurons, which synergistically help to rebuild the local circuitry and allow the electrical signals to be conducted through the injured site^90^, this could be supported by the histoimmunostaining of the spinal cord slices with vGLUT antibodies. Morphologically, the transplanted neurons appeared relatively mature with complex neuritis, which had been further confirmed by determining their indeed firing action potentials and producing synapse current, implying their synaptic formation with endogenous neuronal cells. On the other hand, the WGA tracing system data also provided strong evidence for neuronal relay formation between the transplanted hADSC-MNs and the endogenous neurons.

**Fig. 6:**
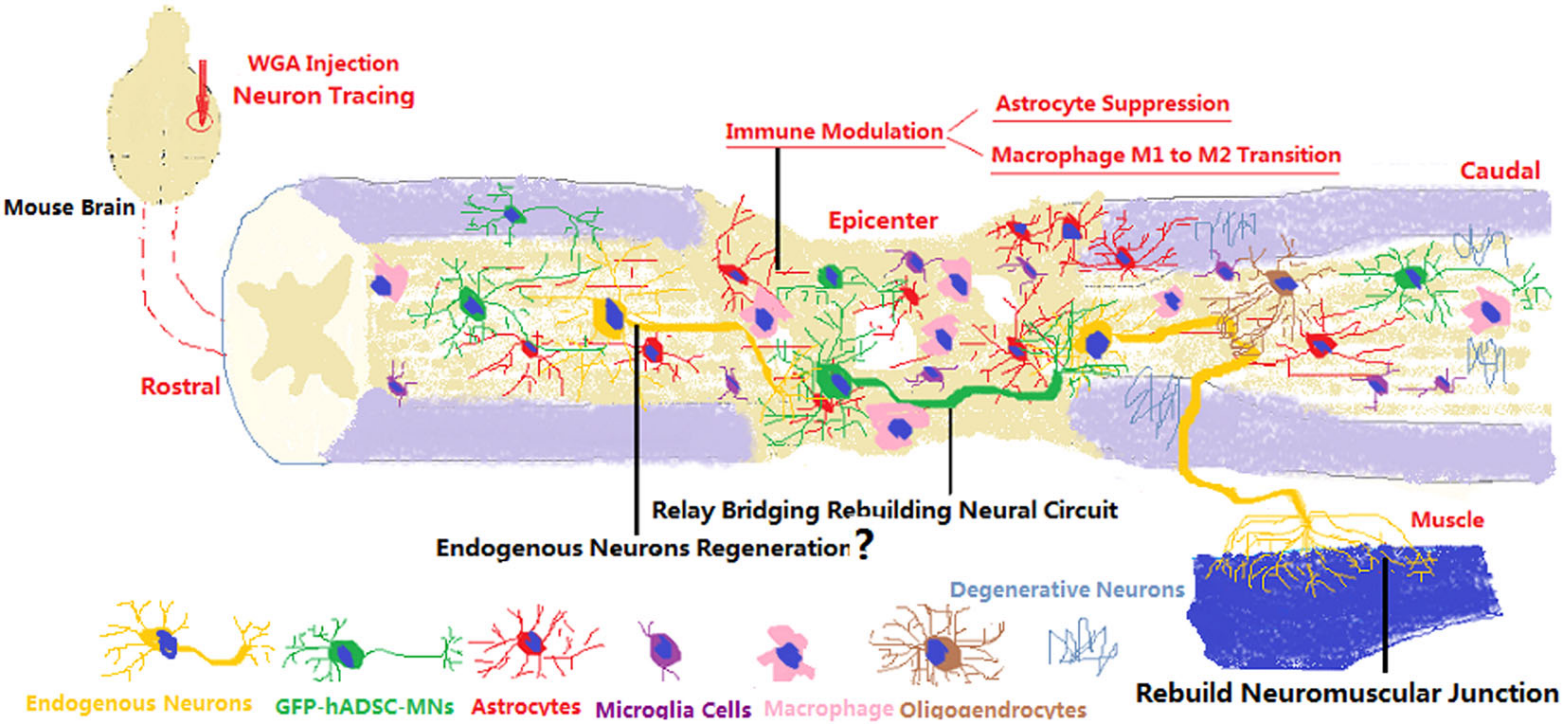
Transplanted hADSC-MNs exert their therapeutic effects through rebuilding neural circuit, immune/inflammatory modulation, and probable promoting endogenous neuron regeneration and in SCI mouse models

This study demonstrated that hADSCs could be transdifferentiated into electrophysiological functional hADSC-MNs expressing motoneuron markers. After being transplanted into the mouse injured spinal cord, significant behavioral recovery was observed. These hADSC-MNs were immune privileged and survived over 8 week. The majority of these hADSCs became neurons and likely participated in rebuilding the damaged circuitry at the site of spinal cord injury by relay mode and optimizing the microenvironment by modulating the postinjury inflammatory response (Fig. 6). Our study not only has provided relative simple strategies to guide the motor neuron differentiation of ADSCs both in vitro and in vivo, has also shown promising therapeutic efficacy of hADSC-MNs in a rodent SCI model.

## Materials and methods

### Differentiation of hADSCs into motoneuron-like cells

hADSCs were washed with PBS at P3 and switched to the serum free motoneuron induction DMEM/F12 medium with the addition of 1 μM purmorphamine or 10 ng/mL SHH and 0.1 μM RA (all trans-retinoic acid)^91^. The detailed protocol referred to the Supplemental information.

### Clinic-relevant mouse SCI compression model

Six- to eight-week-old C57/BL6 mice were housed in a controlled SPF level environment (12 h light/dark cycling at 21 °C) with free access to water and standard chow diet. The crushing protocol referred to the Supplemental information^59^.

### hADSC labeling, motoneuron induction, and transplantation

hADSCs were infected with EGFP-expressing lentivirus FG12 were subjected motoneuron induction, transplantation as the detailed protocol described in the Supplemental information.

### Electrophysiological recording for hADSC-MNs both in vitro and in vivo

To characterize the hADSC-MNs, whole-cell patch-clamp recording was used to assess their electrophysiological activity. The protocols used were as previously described^92–94^. The detailed protocol referred to the Supplemental information.

### Behavioral and histological analysis

Functional tests were performed and analyzed by two examiners blinded to the treatment groups. These tests were performed immediately after the SCI surgery, every 3–4 days for the first 2 weeks after cell transplantation and then weekly for the rest of the 8 weeks. Locomotor function was evaluated using the BMS locomotor rating scale^95^. At the end of 8 weeks, the spinal cord was collected, fixed, postfixed, dehydrated with sucrose, embedded by OCT gel (Sakura Finetechnical), and stored at −80 °C until sectioning. Slices were set as 20 μm and immunohistological staining was carried out as previously described^76^.

### HSV-TK-mCherry-GCV cell ablation system construct and application

HSV-TK-mCherry packaging system was donated by Prof. Zhang Hongsheng. This system was firstly used to infect the in vitro hADSCs to determine the GCV amount for in vivo i.p injection. Six weeks after HSV-TK-mCherry-hADSC-MN transplantation, the transplanted SCI mice received an 7-day i.p injection of GCV at 100 ng/kg body weight per day in order to let the introduced cells commit suicide^96^. The BMS scoring and MEP were performed before and after GCV injection in each group^97,98^. The detailed protocol referred to the Supplemental information.

### Tracing of hADSC-MNs in SCI mice by histoimmunostaining, retrograde neuron tracing manners of WGA

Two weeks before the mice were sacrificed for histoimmunostaining or histochemistry analysis, WGA overexpressing lentivirus (5 µL at 10^9^/mL, the construction data of Lentivirus-WGA refer to Supplementary Fig. 4) were injected into the somatosensory area through a glass micropipette and stereotaxic injector (KDS310, Muromachi-Kikai).

### Immune modulation of hADSC-MNs on spinal cord of SCI mice

To check whether the introduced hADSC-MNs play any immune modulatory roles, 32 inflammation or immune related factors were set in one 96-well panel named mouse cytokine/chemokine magnetic bead panel. The protocols strictly followed the kit (MCYTOMAG-70K-PX32, Lot#2618731) instructions. The experiment performance service was professionally provided by Merck Millipore.

### Statistical analyses

All data are expressed as mean ± SD or SEM. The statistical significance was determined using one-way or two-way ANOVA Bonferroni post-tests between groups by GraphPad Prism version 5.00 for Windows, GraphPad Software, San Diego, CA, USA, www.graphpad.com. *P* < 0.05 was considered statistically significant.

## Supplementary information

Supplemental Manuscript

Fig.Suppl.1

Fig.Suppl.2

Fig.Suppl.3

Fig.Suppl.4

Fig.Suppl.5
