# Supplementary material for: Differentiation of human adipose-derived stem cells into neuron/motoneuron-like cells for cell replacement therapy of spinal cord injury
Source: Cell Death Dis. 2019 Aug 8;10(8):597. doi: 10.1038/s41419-019-1772-1 (PMC6687731; doi:10.1038/s41419-019-1772-1)
Supplement: Supplementary file 1 — Supplemental Manuscript [file 41419_2019_1772_MOESM1_ESM.docx]

**Differentiation of human adipose-derived stem cells into neuron/motoneuron-like cells for cell replacement therapy of**

**spinal cord injury**

Shane Gao^1^, Xuanxuan Guo^1^, Simeng Zhao^2^, Yinpeng Jin^3^, Fei Zhou^4^, Ping Yuan^5^, Limei Cao^6^, Jian Wang^1^, Yue Qiu^1^, Chenxi Sun^1^, Zhanrong Kang^7^, Fengjuan Gao^8^, Wei Xu^5^, Xiao Hu^5^, Danjing Yang^1^, Ying Qin^1^, Ke Ning^9^, Pamela J. Shaw^9^, Guisheng Zhong^#2^, Liming Cheng^#5^, Hongwen Zhu^#10,11^, Zhengliang Gao^#12^, Xu Chen*^6^ , Jun Xu^#1^

^1^ East Hospital, School of Medicine, Tongji University, Shanghai 200120, China

^2^ iHuman Institute, Shanghai Science and Technology University, Shanghai, 201210 , China

^3^ Shanghai Public Health Clinical Center, Fudan University, JinShan, Shanghai,201508, China

^4^ Department of Neurology, Third Affiliated Hospital of Navy Military Medical University, Shanghai, 200438, China

^5^ Tongji hospital affiliated to Tongji University, Tongji University School of Medicine, Shanghai, 200065, China

^6^ Shanghai Eighth People’s Hospital Affiliated to Jiangsu University, Shanghai, 200233, China

^7^ Department of Orthopaedics, Shanghai Pudong Hospital, Fudan University Pudong Medical Center, Shanghai, 200137,China

^8^ Zhoupu hospital, Affiliated to Shanghai University of medicine & health sciences, 201318, China

^9^ Department of Neuroscience, Sheffield Institute for Translational Neuroscience (SITraN), University of Sheffield, 385A Glossop Road, Sheffield S10 2HQ, UK

^10^ Tianjin Hospital, Tianjin 300211, China

^11^ BOE Technology Group Co., LTD, Beijing 100176, China

^12^ Tenth People's Hospital, School of Medicine, Tongji University, Shanghai 200092, China

^#^corresponding author

**METHODS**

**Isolation, expansion and characterization of hADSCs**

Primary hADSCs were extracted from the fresh abdomen liposuction fluid. Briefly, this liposuction fluid was centrifuged at 1000 rpm/min and washed with sterile PBS twice. The pellets were suspended and incubated in 0.075% collagenase type I (Sigma Company) for 30 min at 37 °C, neutralized with 10% FBS-DMEM/F12 medium, centrifuged at 1000 RPM for 10 min and filtered through a 100 μm nylon filter (Falcon Company) The filtrate was centrifuged at 1500 rpm/min and then resuspended and cultured in 10% FBS-DMED/F12 medium (Chemicon). After 5 to 7 days when the cells reached 70~80% confluency, they were trypsinized with 0.05% trypsin/0.5 mM EDTA) and passaged for 2 to 5 generations. To characterize the isolated hADSCs, mesenchymal stem cell markers including CD29 (eBioscience, 11-0299), CD44 (eBioscience, 17-0441-81), CD105 (eBioscience, 12-1057-42) and the hematopoietic stem cell marker CD45 (eBioscience, 11-9459-41), CD133 (Biorbyt, orb4216) were analyzed by immunostaining. Embryonic stem cell markers including SOX2 (Santa cruz, sc-17320), OCT 3/4 (Santa Cruz, sc-9081), c-MYC (Santa Cruz, sc-764) and NANOG (Santa Cruz, sc-30331) were also analyzed by immunocytochemistry and RT-PCR. The primer pairs are listed in Table 1. Human embryonic stem cell cDNA served as the positive control.

**Multi-lineage differentiation experiment.**

The multi-lineage differentiation potential toward adipocytes, osteoblasts, chondrocytes and neuron like cells was performed according to literature. Briefly, for adipocyte induction, we added a cocktail medium including DMEM/F12, 10% FBS, 0.5 mM isobutyl-methylxanthine (IBMX), 1μM dexamethasone, 10μM insulin and 200μM indomethacin. For osteoblast induction, we add a cocktail medium including DMEM/F12, 10% FBS, 0.1μM dexamethasone, 50μM ascorbate-2-phosphate and 10 mM β-glycerophosphate. For chondrocyte induction, we added a cocktail medium including DMEM/F12, 1% FBS, 6.25 μg/ml insulin, 10 ng/ml TGF-β1 and 50 nM ascorbate-2-phosphate. For neuronal induction, we used induction medium composed of DMEM/F12, 200μM butylated hydroxyanisole, 5 mM KCl, 2μM valproic acid, 10μM forskolin 1μM hydrocortisone and 5μg/ml insulin. Induction medium was changed every 3 days. Induction of adipocyte, osteoblast and chondrocyte took 14 days while neuronal induction required only 3-5 days. Oil Red O (Sigma, Cat. No.O0625-25G) staining was used to assess adipogenesis. Alizarin Red S (Sigma, Cat. No. A5533) was used for osteoblast cells and toluidine blue (BIO BASIC INC. Cat. No. TB0961-1G) for chondrocytes.

**Motor Neuron differentiation from hADSC in vitro**

3 days after plating, cells were switched to fresh motoneuron induction medium with 10ng/ml of the neurotrophic factors BDNF, NGF, GDNF and IGF-1 and continued for another 3 days. The neuronal markers MAP2, SYNAPSIN1/2, NeuN and the motoneuron markers Sox1, HB9, Islet and ChAT were used to identify the hADSCs-derived motoneuron- like cells. RT-PCR and qRT-PCR were used to analyze the expression of key markers in these motoneuron-like cells. The profiled genes and their primers are listed in Table 2.

hADSCs were infected with EGFP expressing lentivirus FG12 and 48 hrs later were subjected to motoneuron induction with SHH and RA for 24 hrs. These hADSC-MNs were collected by trypsinization and washed with PBS once, then re-suspended in PBS at 1×10^8^ cell/ml. Seven days after SCI surgery, control and experimental mice (n=7) were injected with either PBS or EGFP labeled hADSC-MNs (3μl/site) at the injury site and the lesion epicenters rostrally and caudally (500μm away from lesion site), using a glass micropipette and stereotaxic injector (KDS310; Muromachi-Kikai).

**hADSC labeling, motoneuron induction and transplantation**

hADSCs were infected with EGFP expressing lentivirus FG12 and 48 hrs later were subjected to motoneuron induction with SHH and RA for 24 hrs. These hADSC-MNs were collected by trypsinization and washed with PBS once, then re-suspended in PBS at 1×10^8^ cell/ml. Seven days after SCI surgery, control and experimental mice (n=7) were injected with either PBS or EGFP labeled hADSC-MNs (3μl/site) at the injury site and the lesion epicenters rostrally and caudally (500μm away from lesion site), using a glass micropipette and stereotaxic injector (KDS310; Muromachi-Kikai).

**Electrophysiological Determination**

Briefly, cells were visualized with a 63× water immersion lens on a Nikon MODEL ECLIPSE FN1 upright microscope (Nikon, Japan) equipped with differential interference contrast (DIC) optics. Patch electrodes with a resistance of 4-7MΩ for somatic recordings were pulled from KG-33 glass capillaries (inner diameter, 1.0mm; outer diameter, 1.5mm; Garner Glass, Claremont, CA) using a P-97 electrode puller (Sutter Instruments CO.). Cells with a seal resistance <5GΩ and a holding current more than -200pA were rejected. The hADSC-MNs were patched in either the voltage-clamp or the current-clamp configuration. The pipette solution for whole-cell recordings contained 123.0mM K-gluconate, 10mM KCl, 1.0mM MgCl_2_, 10.0mM HEPES, 1.0mM EGTA, 0.1mM CaCl_2_, 1.0mM K_2_ATP, 0.2mM Na_4_GTP, and 4.0 mM glucose. The pH was adjusted to 7.2 with KOH. TTX was added into the recording chamber at 1μM by perfusion to block the inward sodium current 5mins after establishing the whole-cell voltage recording. For the recording in live spinal cord, firstly, the spinal cord slices were prepared based on NMDG (*N*-methyl-glucamine) recovery slice methods. Briefly, After anestherization using isoflurane, the mice spinal cord were exposed and the injured sites as well as the surroundings were removed and placed in cold oxygenated NMDG solution (in mM: NMDG 93, KCl 2.5, NaH_2_PO_4_ 1.2, NaHCO_3_ 30, HEPES 20, D-glucose 25, sodium ascorbate 5, thiourea 2, sodium pyruvate 3, MgSO_4_ 10, CaCl_2_ 2, HCl 93, pH 7.35). The spinal cord were cut into thin slices (150 μm) using a vibratome (Leica VT1200S, Germany), the slices were incubated in oxygenated NMDG solution at 37 ^o^C for 15mins and then transferred to the physiological solution (in mM: NaCl 125, KCl 2.5, NaH_2_PO_4_ 1.25, NaHCO_3_ 25, D-gluocose 25, MgCl_2_ 1, CaCl_2_ 2, pH 7.4) and incubated for 0.5 – 1 h before recording. A whole cell patch clamp technique was then used. One spinal cord slice was transferred into the recording chamber, continually perfused with oxygenated artificial cerebrospinal fluid (ASCF, in mM: sucrose 213, KCl 2.5, NaH_2_PO_4_ 1.25, NaHCO_3_ 26, D-glucose 10, MgSO_4_ 2, CaCl_2_ 2, pH 7.4) and viewed under a DIC microscope (60 × water immersion lens, Olympus, Japan). The electrophysiological properties of the cells with green fluorescence were recorded using a HEKA amplifier (HEKA EPC 10 USB, Germany). The electrode puller (Sutter P-1000, USA) was used to make electrodes with the resistance at 10 – 13 MΩ when filled with the neurobiotin tracer (1 mM, Vectorlabs, USA) contained pipette solution (in mM: potassium gluconate 140, KCl 3, MgCl_2_ 2, HEPES 10, EGTA 0.2, Na_2_ATP 2, pH 7.25).

**Spinal Cord Injury mouse model**

All mice were pre-conditioned in the surgical room for 2 hrs before anesthetization with 2% (0.15ml/10g mouse body) of chloral hydrate (C_2_H_3_Cl_3_O_2_). Under dissection microscopy, the mouse skin was incised to remove the T 7vertebral arch and expose the spinal cord. Modified fine-tip forceps with 0.4 mm spacer were used to compress the cord laterally from both sides for 10s. After suture, mice were kept warm until recovery from anesthesia. Hindlimb locomotor function was then tested by the Basso Mouse Scale (BMS) method. Mice with a successful SCI surgery defined by a BMS score of 0-3 were included in subsequent experiments.

**Motor-evoked potentials (MEP) detection for neural circuitry re-building**

Briefly, all transcranial magnetic motor-evoked potential (tcMMEP) recordings were obtained from awake, nonanesthetized, restrained mice. The active electrode needle was inserted into the belly of the gastrocnemius muscle, a second reference electrode needle was inserted near the distal tendon of the gastrocnemius muscle, and a third ground electrode needle was inserted into the base of the tail. tcMMEPs were elicited using a magnetic stimulator (Cadwell Laboratories; Kennewick, WA) with a 5.0-cm doughnut-shaped coil. The center of the coil was placed at a 45ºangle lying on the head of the animal. A single pulse with 100% intensity was repeated three times with a 30-sec lapse between trials bilaterally. Data were collected by Dantec Keypoint software. The onset latency was measured in milliseconds (msec) and the peak-trough amplitude was measured in millivolts (mV). Amplitude background noise was determined to be approximately 40 microvolts (mV); we set the signal-to-noise ratio at four times this level (160 mV). Recordings with amplitudes less than 160 mV were considered noise. The latency window was established to fall between 4 and 5 msec. Baseline recordings were obtained prior to injury.

**HSV-TK-mCherry-GCV cell ablation system construct and application**

We took the advantage of the Herpes Simplex Virus type 1 (HSV-1) thymidine Kinase (TK) overexpression system with mCherry reporter (HSV-TK-mCherry) which could overexpress the viral specific TK and label the hADSC-MNs simultaneously. Once the antiviral drug Ganciclovir (GCV) is i.p injected, it will be transferred into phosphorylated form by TK, which can inhibit DNA synthesis and eventually lead to the apoptosis of the transfected cells. Before transplantation, hADSCs were firstly infected by HSV-TK-mCherry lentivirus and conditioned toward MN differentiation and then transplanted into the spinal cord of the SCI mouse model in the same way as the GFP labeled hADSC-MNs.


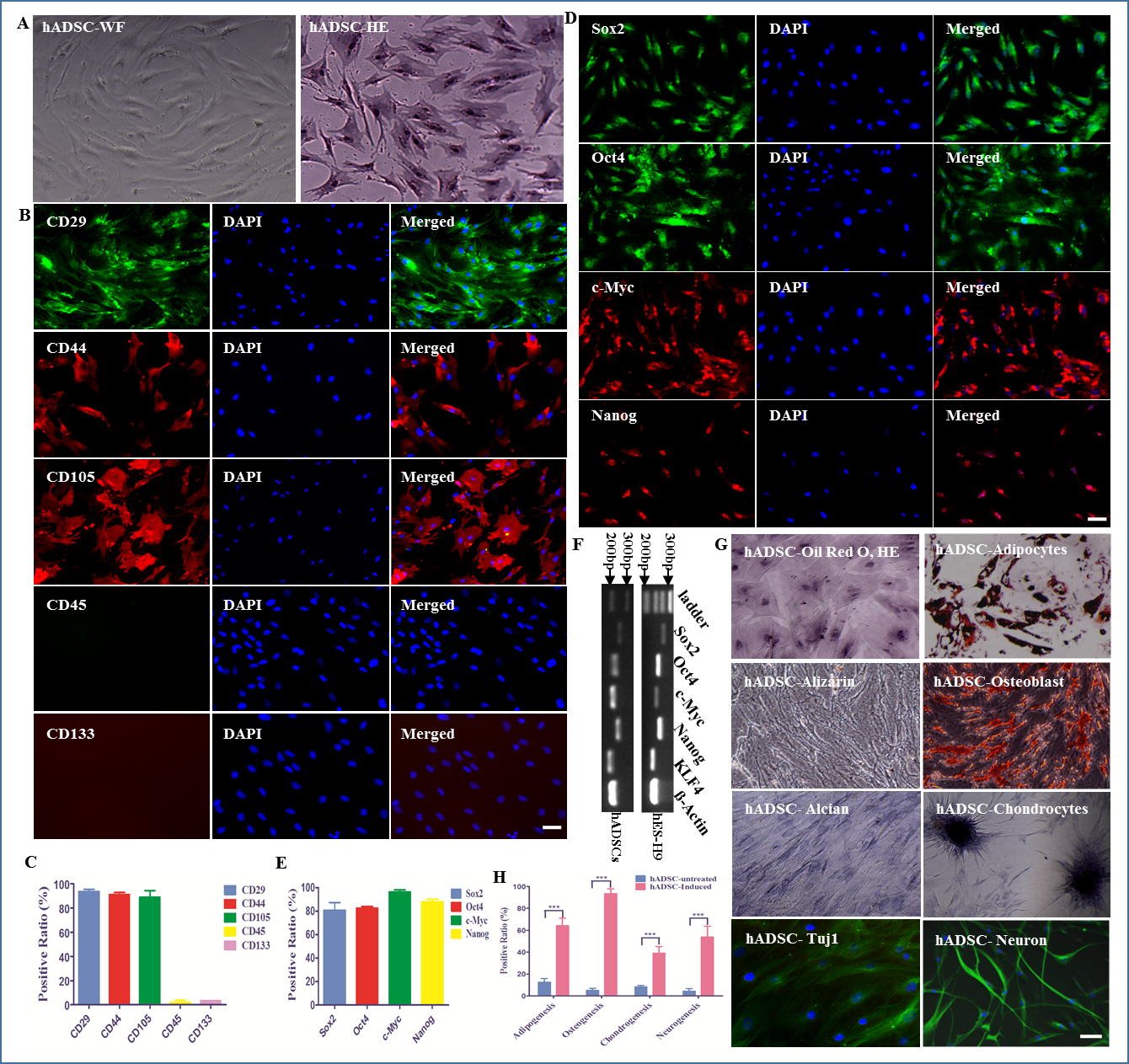


**Figure Suppl.1.Characterization of hADSCs by surface markers, gene marker and multi-potency** (A) hADSCs at P3 grow swirling on plastic dish stained by HE with spindle-like cell morphology; (B) more than 85% of hADSCs at P3 positively express CD29, CD44 and CD105 and less than 5 % of hADSCs express CD45 and CD133; (C) quantification of positive ratio of various markers in hADSCs at P3; (D) hADSCs at P3 also express typical embryonic stem cell(ESC) markers Sox2, Oct4, c-Myc, Nanog, KLF4; (E) quantification of positive ratio of various embryonic stem cell markers in hADSCs; (F) hADSCs express Sox2, Oct4, c-Myc, Nanog and KLF4 genes but at lower level confirmed by RT-PCR compared with ESC cell line H9; (G) hADSCs have the capacity to differentiate into adipocyte (stained with Oil Red O and the nucleolus stained in contrast with hematoxylin eosin (HE)), osteoblast cells (stained with Alizarin red), chondrocytes (stained with Alcian blue) and neuron like cells (stained with Tuj1); (H) the differentiation efficiency is quantified by the positive ratio of various markers. the quantification is made out of at least three independent experiments. Scale bar:100 um.

**Figure Suppl.2. Differentiation of hADSC.s into motoneuron-like cells by a step-wise protocol** (A) Before induction, hADSCs have little expression of neuronal or motoneuron markers such as NeuN, ChAT, Synapsin1/2 except to low expression of MAP2 and HB9; (B) After 3 day induction with Purmorphamine (or SHH) and RA, hADSCs start to express high level of motoneuron progenitor markers Olig2, Sox1, medium level of some neuron/motoneuron MAP2, Synapsin1/2, HB9, ChAT, Islet and NF-200; (C) Another three days’ induction supplemented with neurotrophic factors of BDNF, NGF, GDNF, IGF, further boosts the maturation of the hADSC derived motorneuron-like cells with the high level expression of neuron/motoneuron markers MAP2, NeuN, NF-200, Synapsin1/2, Islet, HB9 and ChAT, meanwhile the motoneuron progenitor markers expression decreased. The neuritis of the differentiated cells become more complex; (D) quantification of immunostaining for various markers at different stages; Sc
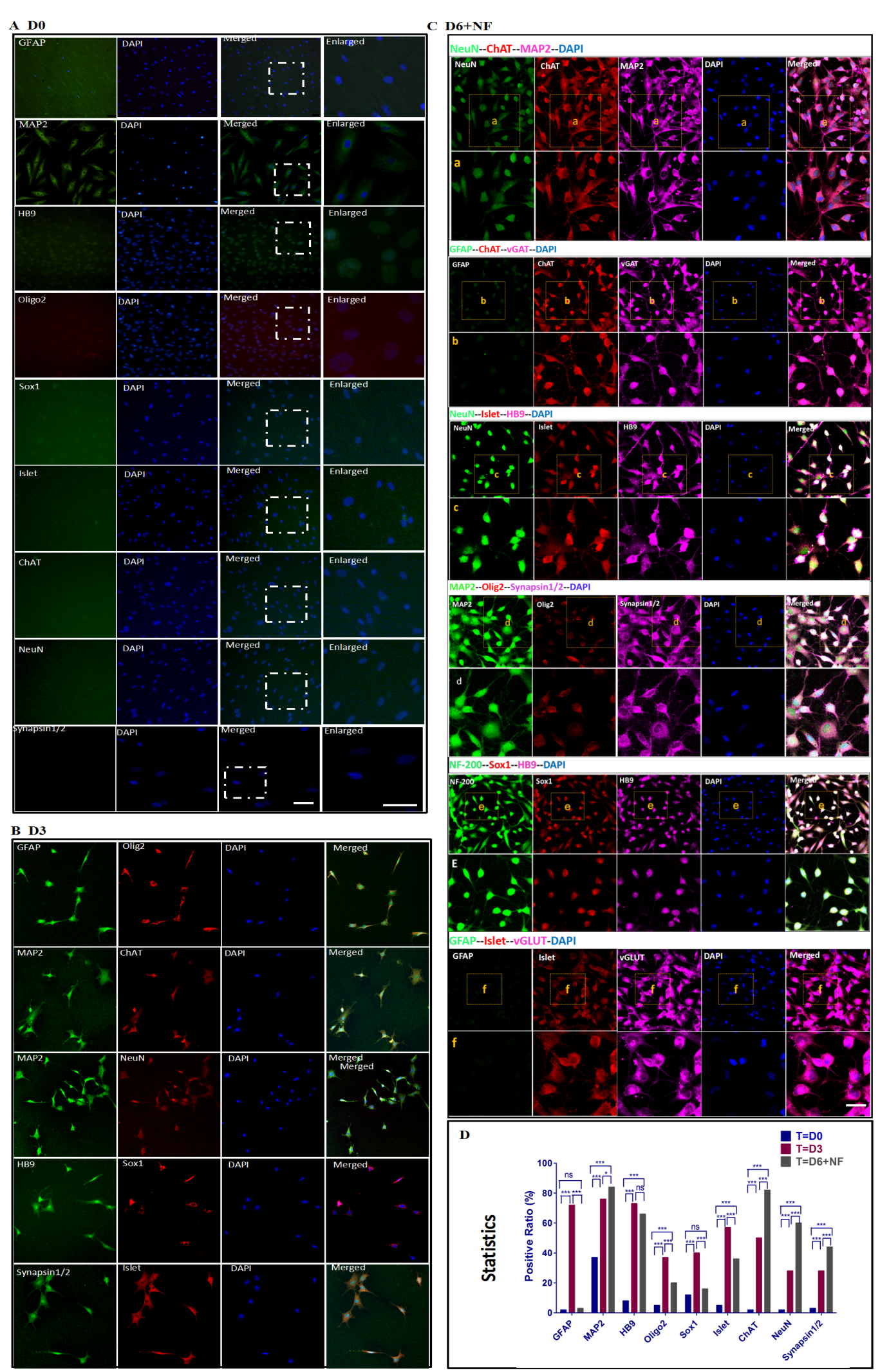
ale bar:100 um. Statistics is done by two-way ANOVA of Graphpad Prism 5.


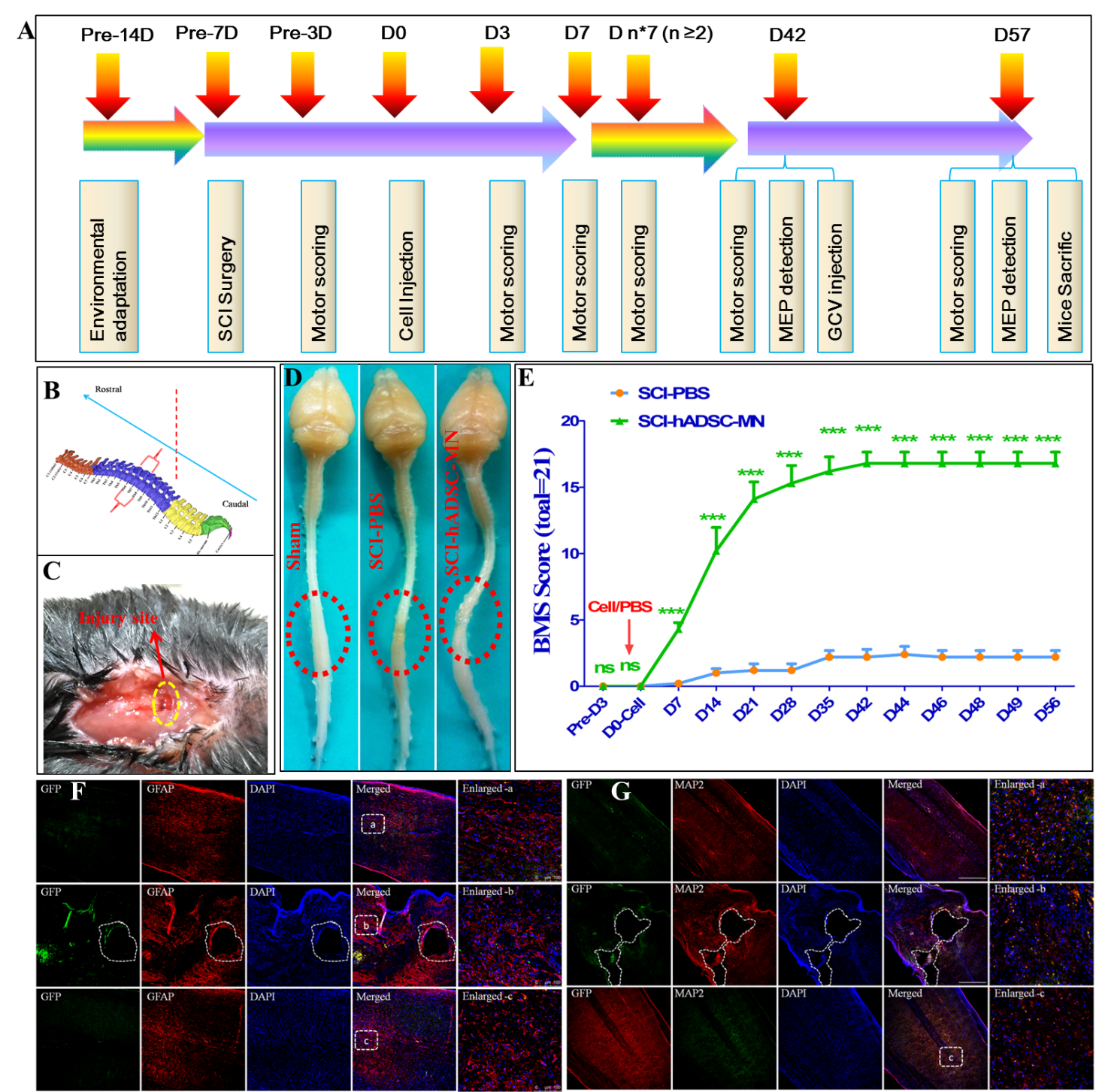


**Figure Suppl.3.** Transplanted hADSC-MN promotes their hindlimp locomotor function od the spinal cord injury mice (A) Schematic experimental procedure was showed by the schematic flow chart; (B) Forcing injury at T8 was performed strictly at SPF conditions under anesthesia; (C) The yellow dash circled the dark grey line shows the successful SCI surgery; (D) 8 weeks after hADSC-MN cells transplantation, the spinal cord tissue repair can be observed; (E) BMS scores in total including the primary- and sub-score were recorded. hADSC-MN cells were injected 7 days after SCI surgery, meanwhile SCI injected with PBS serving as the control group (n=12); GCV was i.p. injected every day from D49 till D56; (F&G) IHC for SCI-PBS group slices using GFAP and MAP2 antibodies show appearance of relatively larger injury cavities, reaction of astrocytes and non GFP and MAP2 double positive cells.; (F&G) Histoimmunostaining of the spinal cord cryosection with GFAP and MAP2 for the untreated PBS group of spinal cord injury mice. Scale bar:100um.


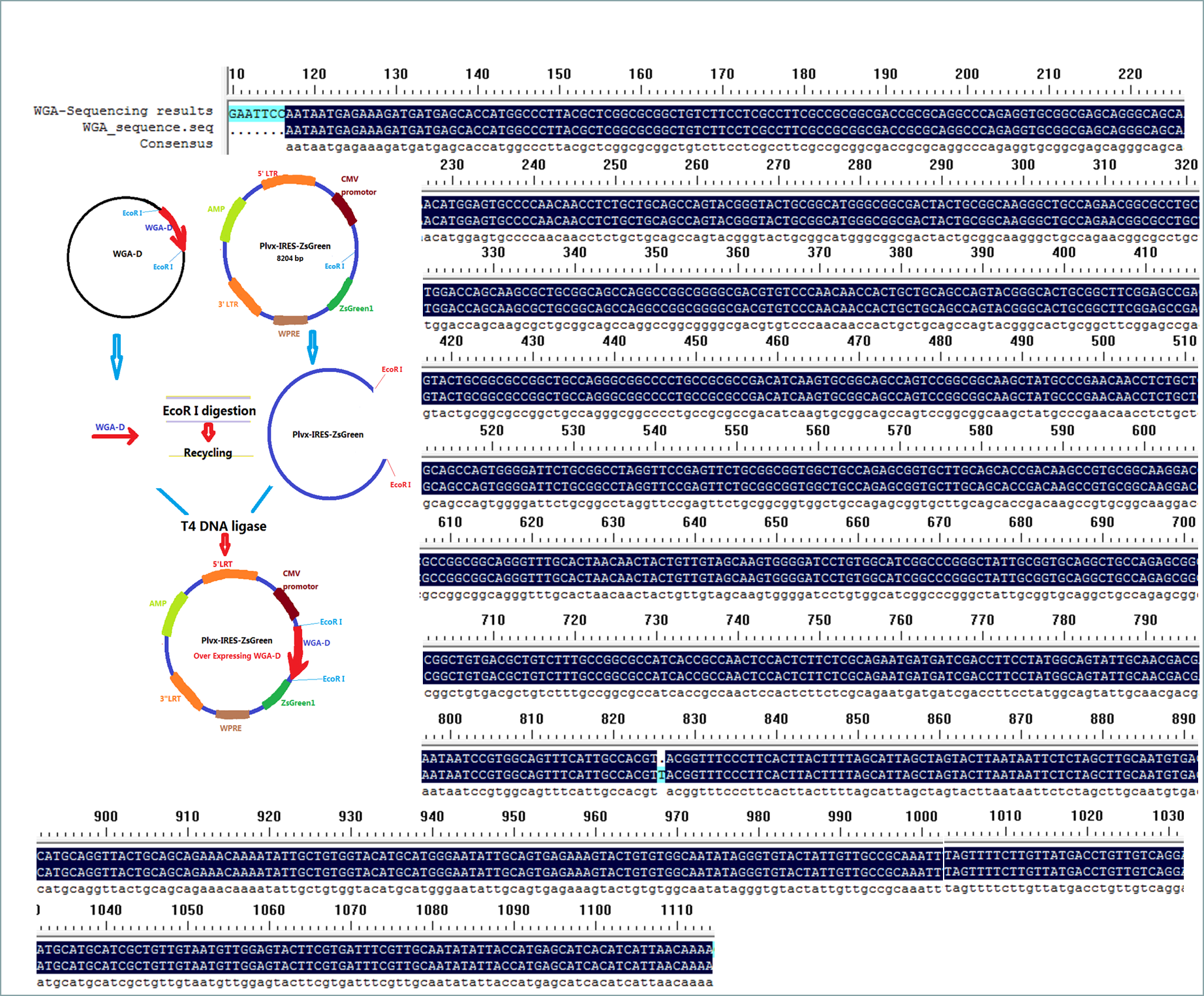


**Figure Suppl.4. WGA cloning onto the Plvx-IRES-ZsGreen and the sequencing results.** The schematic working flow chart of WGA gene cloning was shown. WGA-D fragment (782bp) harbored by the pBKS (Plasmid #17989) was bought from addgene ([http://www.addgene.org](http://www.addgene.org/)), amplified and digested by EcoR I. Then the WGA-D fragment was subcloned onto the Plvx-IRES-ZsGreen and sequenced by the company. Sequencing results was aligned with the WGA sequence by DNA man and shown above.


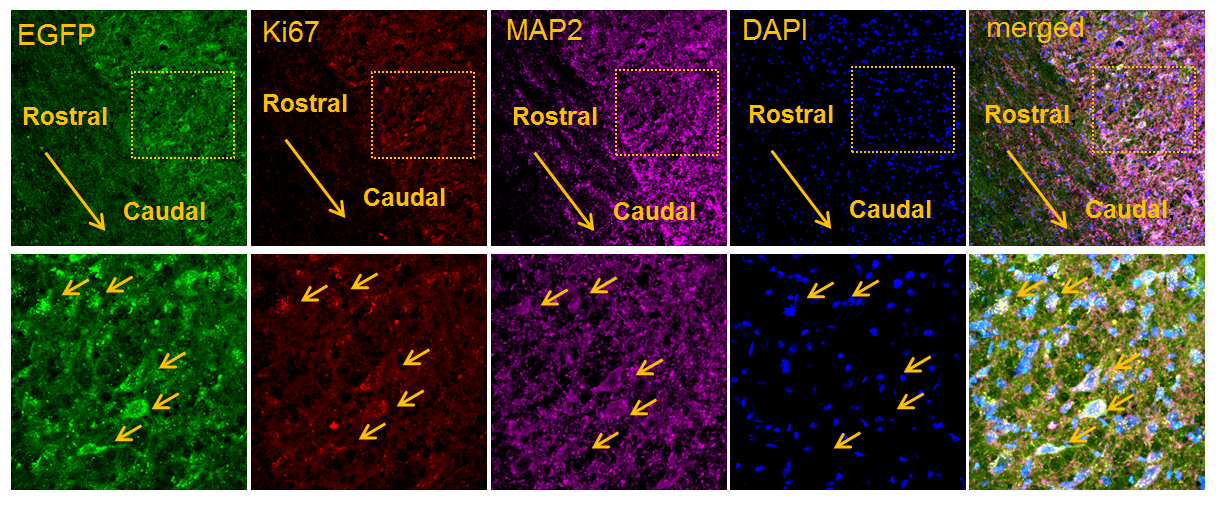


**Figure.Suppl.5 Using Ki67 antibody to determine whether the transplanted EGFP-labled hADSC-MNs still possess proliferation ability.** Some of them are Ki67 positive with minor MAP2 expression as the yellow arrows show , while others are Ki67 negative but MAP2 positive, indicating with time lapse, after the hADSC-MNs obtain their neuronal properties, their proliferation ability will disappear.

**Video list**

SCI-D1-MN-1#

SCI-D7-MN-1#

SCI-D49-MN-1#

SCI-D56-MN-1#

SCI-D**-MN-1#

means spinal cord injury treated with hADSC derived motor neuron like cells on day **

SCI-D1-PBS-1#

SCI-D7-PBS-1#

SCI-D49-PBS-1#

SCI-D56-PBS-1#

means spinal cord injury treated with PBS on day ** for animal as the negative control

Sham

Means the Sham control without compression of spinal cord
