# Supplementary figures and images for: Differentiation of human adipose-derived stem cells into neuron/motoneuron-like cells for cell replacement therapy of spinal cord injury
Source: Cell Death Dis. 2019 Aug 8;10(8):597. doi: 10.1038/s41419-019-1772-1 (PMC6687731; doi:10.1038/s41419-019-1772-1)

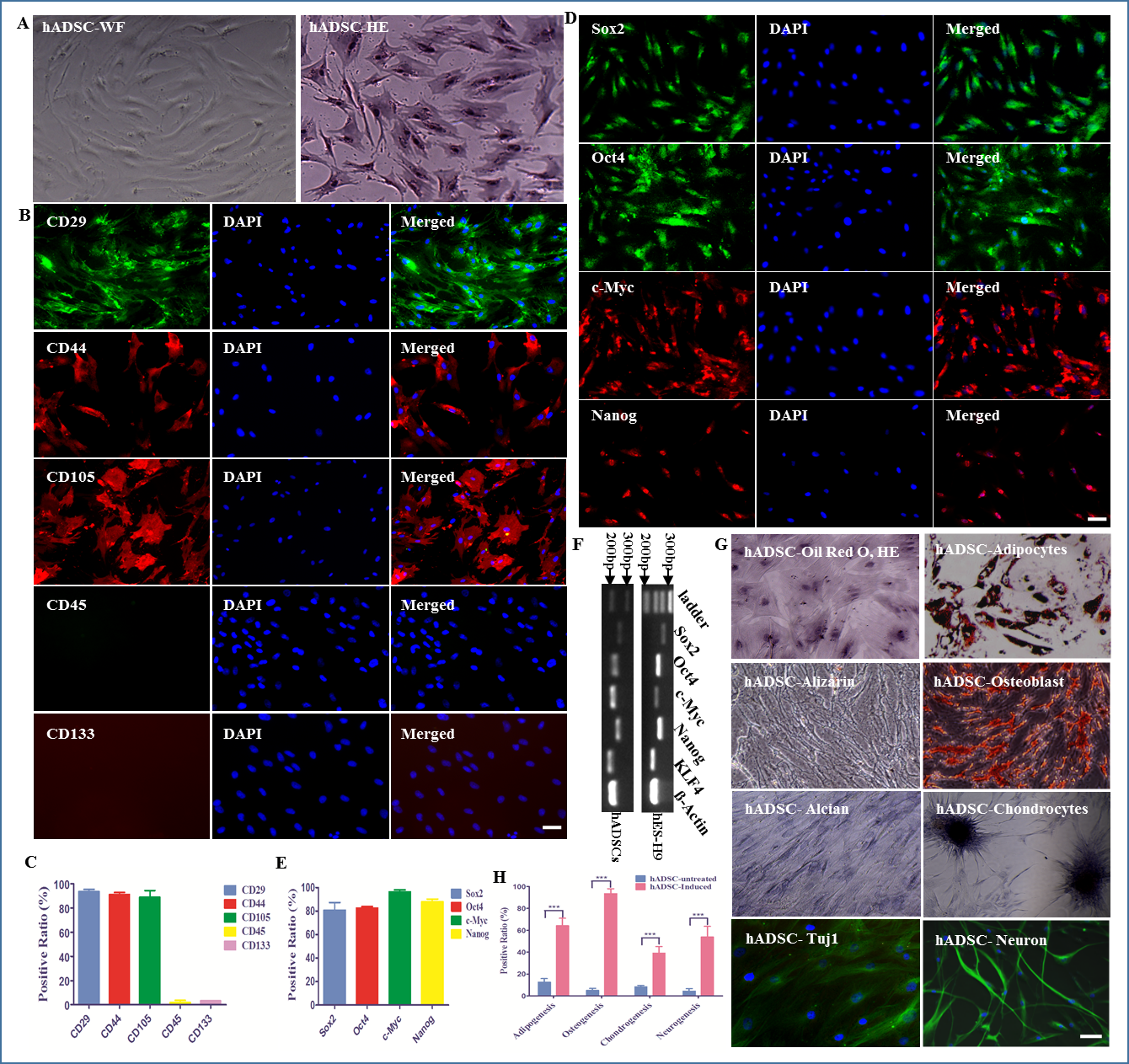

Supplement: Supplementary file 2 — Fig.Suppl.1 [file 41419_2019_1772_MOESM2_ESM.tif]

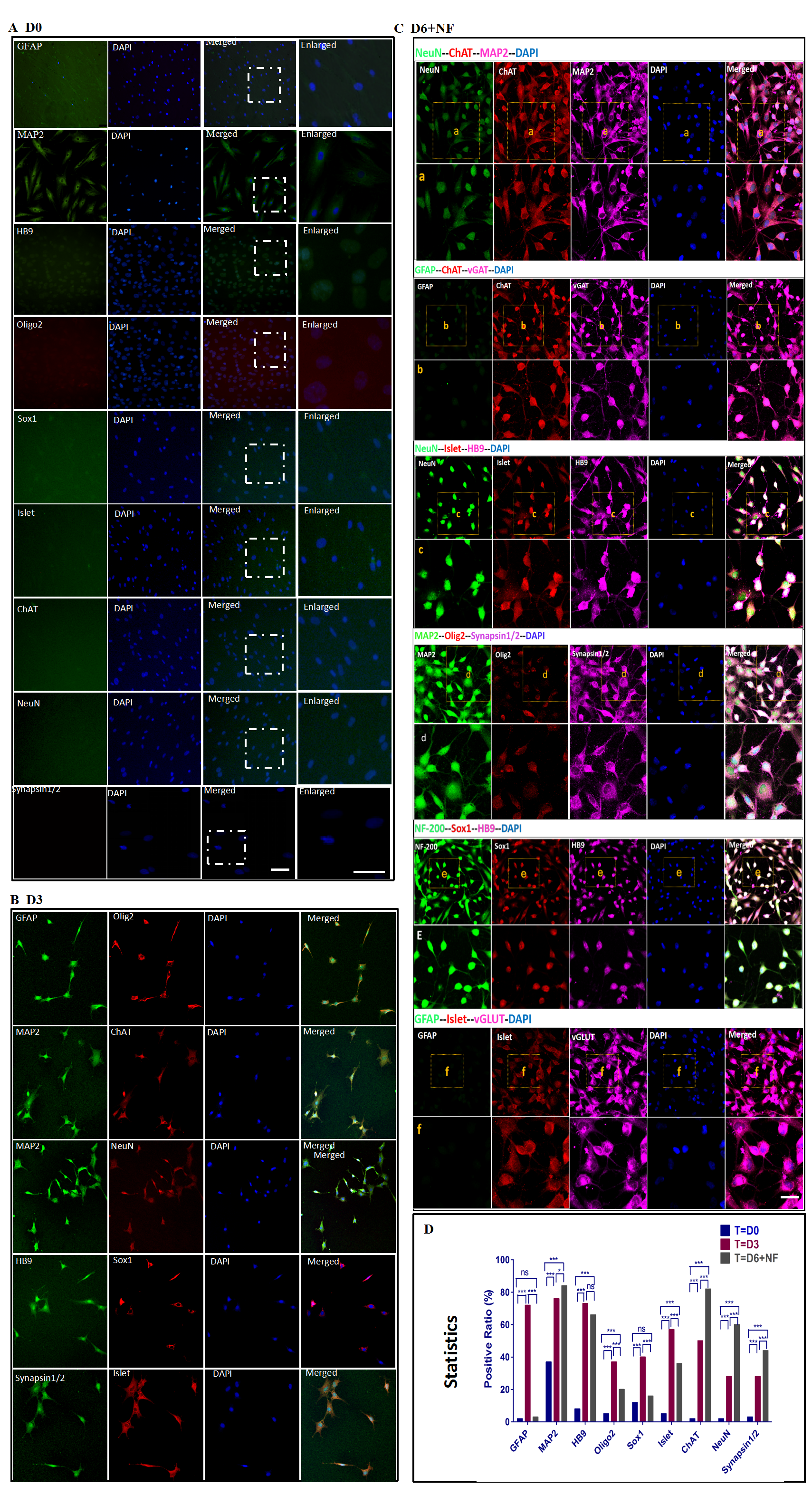

Supplement: Supplementary file 3 — Fig.Suppl.2 [file 41419_2019_1772_MOESM3_ESM.tif]

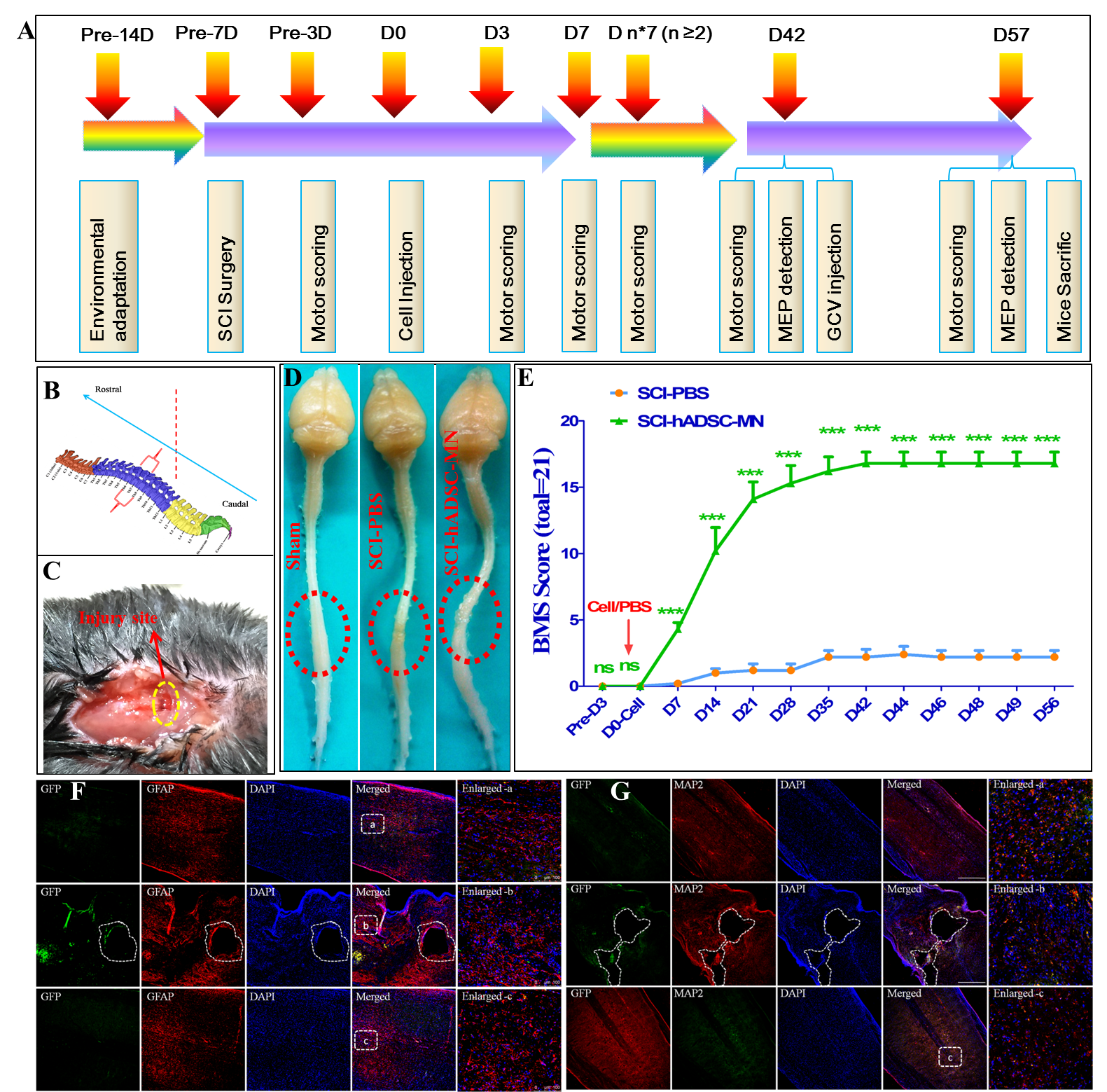

Supplement: Supplementary file 4 — Fig.Suppl.3 [file 41419_2019_1772_MOESM4_ESM.tif]

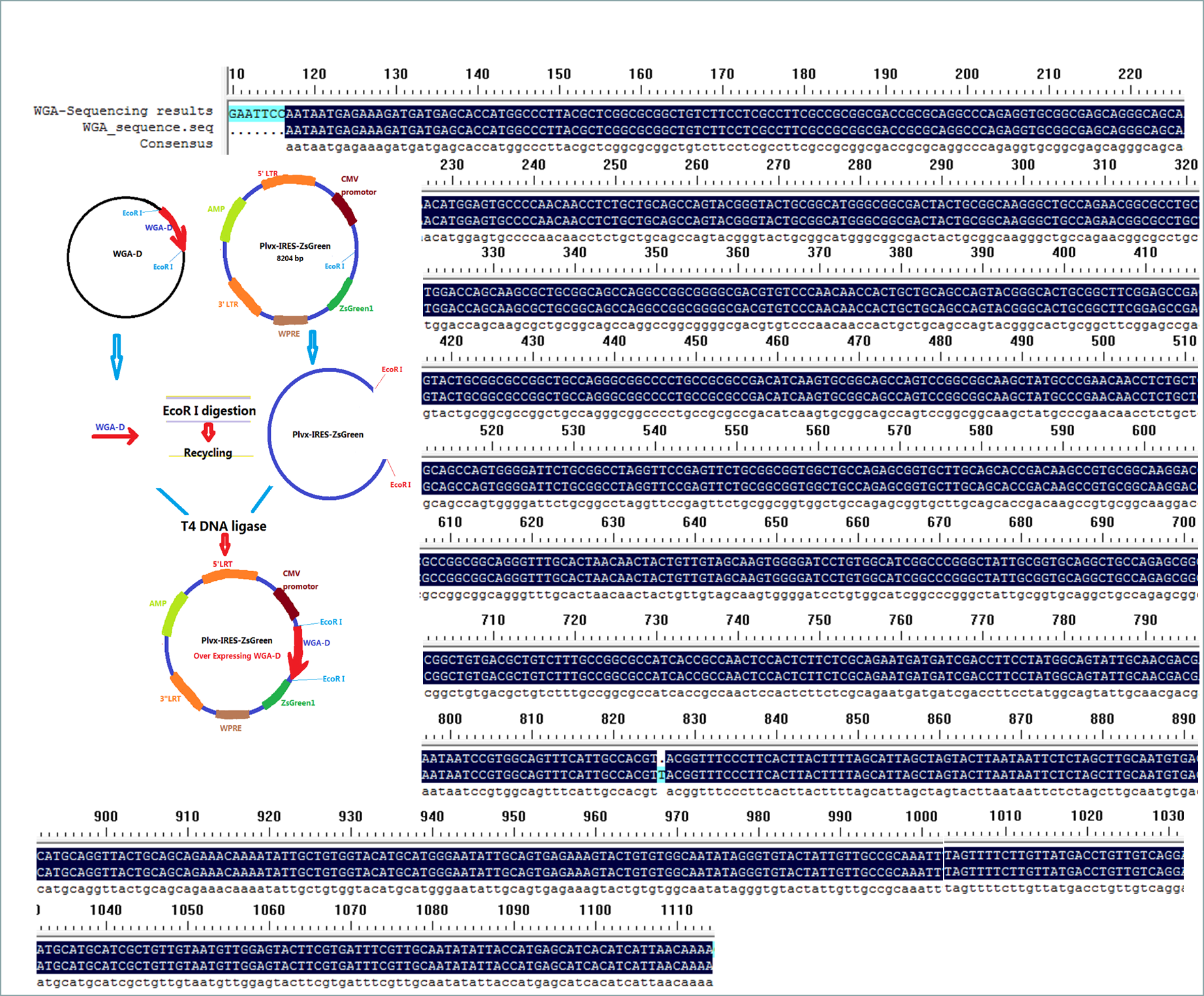

Supplement: Supplementary file 5 — Fig.Suppl.4 [file 41419_2019_1772_MOESM5_ESM.tif]

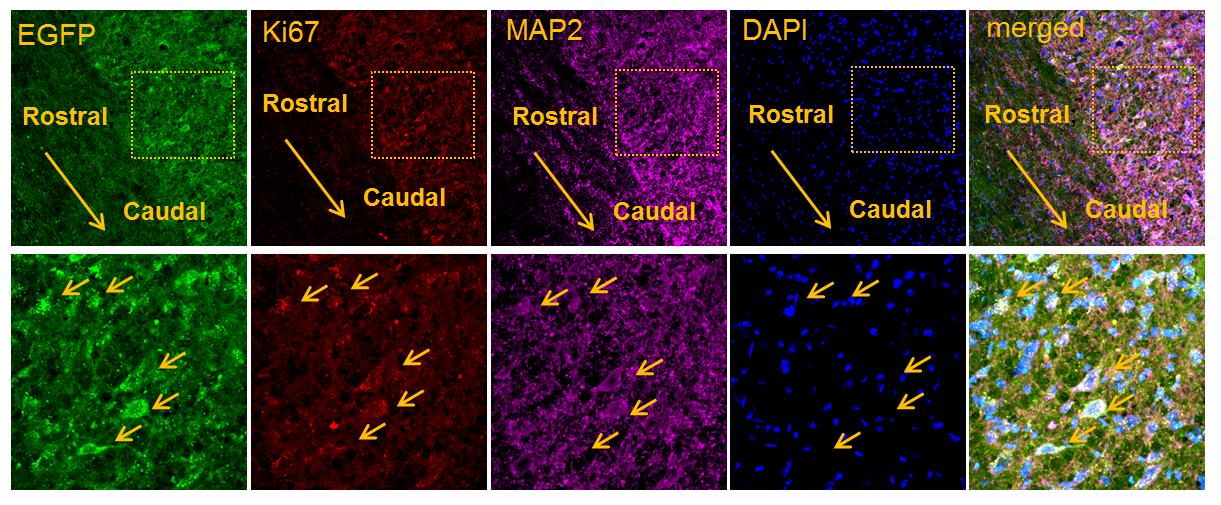

Supplement: Supplementary file 6 — Fig.Suppl.5 [file 41419_2019_1772_MOESM6_ESM.tif]
